# A Decline in Benthic Foraminifera following the Deepwater Horizon Event in the Northeastern Gulf of Mexico

**DOI:** 10.1371/journal.pone.0120565

**Published:** 2015-03-18

**Authors:** Patrick T. Schwing, Isabel C. Romero, Gregg R. Brooks, David W. Hastings, Rebekka A. Larson, David J. Hollander

**Affiliations:** 1 College of Marine Science, University of South Florida, Saint Petersburg, Florida, United States of America; 2 Eckerd College, Saint Petersburg, Florida, United States of America; ufrj, BRAZIL

## Abstract

Sediment cores were collected from three sites (1000–1200 m water depth) in the northeastern Gulf of Mexico from December 2010 to June 2011 to assess changes in benthic foraminiferal density related to the Deepwater Horizon (DWH) event (April-July 2010, 1500 m water depth). Short-lived radioisotope geochronologies (^210^Pb, ^234^Th), organic geochemical assessments, and redox metal concentrations were determined to relate changes in sediment accumulation rate, contamination, and redox conditions with benthic foraminiferal density. Cores collected in December 2010 indicated a decline in density (80–93%). This decline was characterized by a decrease in benthic foraminiferal density and benthic foraminiferal accumulation rate (BFAR) in the surface 10 mm relative to the down-core mean in all benthic foraminifera, including the dominant genera (*Bulimina* spp., *Uvigerina* spp., and *Cibicidoides* spp.). Cores collected in February 2011 documented a site-specific response. There was evidence of a recovery in the benthic foraminiferal density and BFAR at the site closest to the wellhead (45 NM, NE). However, the site farther afield (60 NM, NE) recorded a continued decline in benthic foraminiferal density and BFAR down to near-zero values. This decline in benthic foraminiferal density occurred simultaneously with abrupt increases in sedimentary accumulation rates, polycyclic aromatic hydrocarbon (PAH) concentrations, and changes in redox conditions. Persistent reducing conditions (as many as 10 months after the event) in the surface of these core records were a possible cause of the decline. Another possible cause was the increase (2–3 times background) in PAH’s, which are known to cause benthic foraminifera mortality and inhibit reproduction. Records of benthic foraminiferal density coupled with short-lived radionuclide geochronology and organic geochemistry were effective in quantifying the benthic response and will continue to be a valuable tool in determining the long-term effects of the DWH event on a larger spatial scale.

## Introduction

The Deepwater Horizon (DWH) event released over 4.9 million barrels of oil into the Gulf of Mexico from April to July of 2010 [1]. An estimate of 60% of the oil reached the surface where it was subject to skimming, coastal deposition, evaporation, and incorporation into flocculent material [2]. Flocculent material consisting of algae, dispersant, clay particles, and microbes formed at the water surface with the aggregated oil and settled to the sea floor [3]. Subsurface intrusions of natural gas and oil also formed in the water column, with the dominant intrusion occurring from 1000–1300 m predominantly along a northeast to southwest transect [4,5]. Ryerson et al. [6] estimated that only 35% of the oil made it to the water surface and 35% was included in the subsurface intrusion. The estimates from Ryerson et al. [6] and Thibodeaux et al. [2] suggest that as much as 30–40% of the oil unaccounted for was likely deposited on the seafloor.

There are several pathways for oil to be transported to the seafloor. The two primary hypotheses are: (1) the bathtub-ring hypothesis and (2) the flocculent blizzard hypothesis. The bathtub-ring hypothesis refers to the direct contact of microdroplets and dissolved hydrocarbons from the subsurface intrusion [7] at the sediment-water interface on the continental slope. The flocculent blizzard hypothesis refers to the large amount of organic flocculent and hydrocarbon material (large droplet phase) that was deposited during, and following, the event [8,9].

The study of benthic foraminifera provides several strengths in assessing the effects of the Deepwater Horizon event on the benthic environment. There are high densities of benthic foraminifera in the shelf and slope sediments of the Gulf of Mexico [10–12], which allows for a robust assessment of changes in benthic foraminiferal density. The lifespan of benthic foraminifera is on the order of months to years, which readily allows for adaptation to environmental changes [13,14]. This turnover provides an event stratigraphy of benthic foraminiferal density on the order of an event such as the DWH (several months). Finally, benthic foraminifera are sensitive to the introduction of toxins and hydrocarbons [15–21].

Many studies have described the benthic foraminifera assemblages associated with the shelf and slope environments in the Gulf of Mexico [10,11,22–27]. Bernhard et al. [27] documented a dominance of agglutinated benthic foraminifera at all sampling sites from 500–3000 m water depth. Denne and Sen Gupta [24] identified a specific benthic foraminifera assemblage dominated by *Cibicidoides wuellerstorfi*, *Bulimina aculeata* and others associated with Caribbean Midwater (CMW), which is dominant between 850 and 1500 m water depth. Culver and Buzas [23] identified the dominant benthic foraminifera species in the outer shelf and slope as *Bulimina marginata* and *Uvigerina peregrina*. Osterman [26] also identified *Cibicidoides pachyderma* (epifaunal), *Uvigerina peregrina* (shallow infaunal) and *Bulimina aculeata* (shallow infaunal) as the dominant species in the upper and lower slope sediments. Sen Gupta and Aharon [25] suggested that near hydrocarbon seeps in the northern Gulf of Mexico, several species of benthic foraminifera are possibly facultative anaerobes and can adapt to periods of anoxia and high hydrocarbon concentrations.

**Fig 1:**
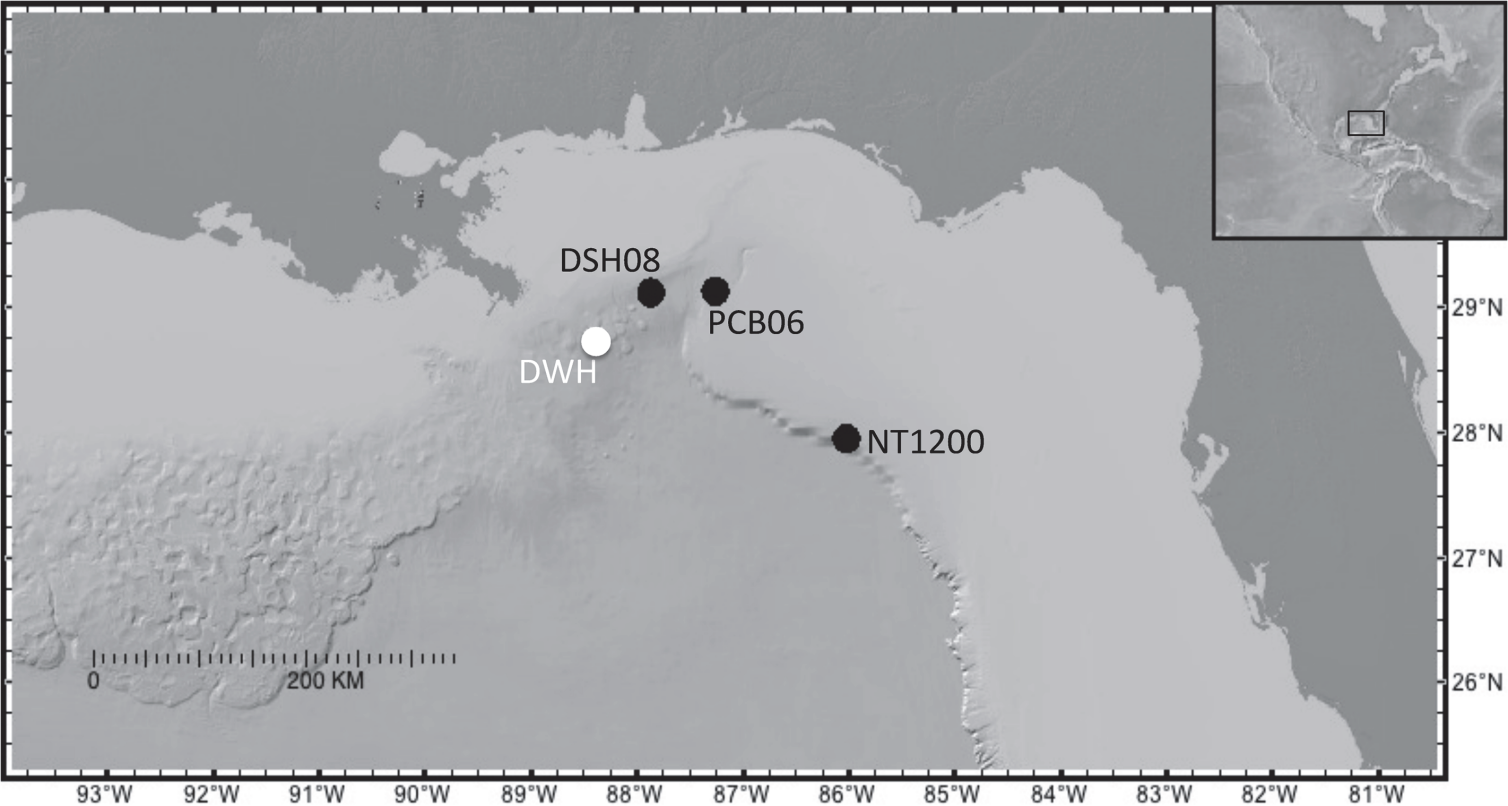
Location of core sampling sites in the northeastern Gulf of Mexico with reference to the Deepwater Horizon.

Models suggest that a considerable amount of oil may have been transported into the Desoto Canyon (Northeastern Gulf of Mexico) during and following the DWH event [7]. Other studies have documented a 4–10-fold increase in sediment accumulation rate [9], persistent reducing (anoxic) zones in the surface sediment [28], and a 2–3-fold increase in polycyclic aromatic hydrocarbons (PAH) concentrations [29] in the Desoto Canyon following the DWH event. The primary objective of this study is to characterize the impacts of the Deepwater Horizon event on the benthic foraminiferal density in sediment cores, collected from 1050–1150 m water depth in the Desoto Canyon after the DWH event (Fig. 1). This study aims to report temporal changes in the benthic foraminiferal density and to further propose the most likely factors that caused those changes. The observation of a decline in benthic foraminiferal density synchronous with other sedimentary and geochemical signatures suggests an impact on the benthic environment following the DWH event.

## Methods

### Field methods

Sediment cores were collected throughout the northeastern Gulf of Mexico (Fig. 1) using an Ocean Instruments MC-800 multicoring system, which collects eight cores simultaneously. At each site, one of the eight cores collected was utilized for benthic foraminifera analysis and was paired with one core utilized for short-lived radionuclide (SLR) geochronology, one core for organic geochemistry, and one core for redox metal chemistry. Two initial sites [PCB06 (29° 5.99’ N, 87° 15.93 W, 1043 m depth) and DSH08 (29° 7.25’ N, 87° 51.93’ W, 1143 m depth)] were chosen for benthic foraminifera analysis due to preliminary organic geochemistry results suggesting the presence of oil (University of South Florida, College of Marine Science’s baseline survey, 29) and each site was located at a water depth that was within the range of the documented primary hydrocarbon plume (1000–1300 m) [1,6]. Due to the lack of records taken pre-DWH in the area of DSH08 and PCB06, a core was collected at site NT1200 (27° 57.98’ N, 86° 1.38’ W, 1200 m depth) to provide a control record representing an area that was outside of the surface and intrusion (plume) expression of elevated hydrocarbons concentrations related to the DWH event. No specific permissions were required to collect sediments at these sites and did not involve any protected or endangered species. Cores were refrigerated (∼4°C) until sub-sampled by extrusion at 2 mm intervals for the upper 50 mm, with the exception of the December 2010 DSH08 core (2mm to 20mm), and 5 mm intervals for the remainder of the core using a calibrated, threaded-rod extrusion device [30,31].

### Benthic foraminifera

Extruded subsamples were freeze-dried, weighed and washed with a sodium hexametaphosphate solution through a 63-μm sieve to disaggregate the clay particles from foraminifera tests. The fraction remaining on the sieve (coarse fraction) was dried, weighed again, and stored at room temperature. All benthic foraminifera were picked from the samples, identified, and counted. Total density is reported, as opposed to living community density for direct comparison of up-core (post-DWH) to down-core (pre-DWH/background) records [26,32,33]. The down-core (pre-DWH) sections were utilized as background samples due to the lack of previous coring efforts at these sites. The use of down-core samples as background samples was based on the assumption that there are statistically negligible numbers of living foraminifera at these depths (e.g. 100 mm) [17,31,34] and that they represent periods of deposition before influence from the DWH event [9]. The total density approach was also appropriate seeing as these records were to be used as reference records when determining any persistent sedimentary (physical, chemical, biological) features related to the DWH event in future sedimentary records on the decadal time-scale [26,32]. Foraminiferal density values were reported in individuals per unit volume (indiv./cm^3^)[34]. The values were normalized to the known wet volume of each sample based on the diameter of the core tube (10 cm) and the height of each sample (2 or 5 mm). Non-metric multi-dimensional scaling (nMDS) plots (Bray Curtis) were constructed using the PAST paleo-statistics suite to assess the relative control of redox metal concentration and PAH concentration on benthic foraminiferal density.

### Short-lived radionuclide geochronology

Extruded subsamples were freeze-dried, weighed, homogenized, and sealed in plastic containers. Short-lived radioisotope geochronology was used to distinguish the pre-DWH and post-DWH intervals. Samples were counted on a Canberra HPGe (high-purity germanium) coaxial planar photon detector to determine ^210^Pb and ^234^Th activity. Activities were corrected for counting time, detector efficiency, and self- absorption using the IAEA RGU-1 standard [35, 36]. The constant rate of supply (CRS) model was employed to assign a date-depth relationship, which is appropriate under varying accumulation rates [37, 38].

### Benthic foraminiferal mass accumulation rates (BFAR)

Considering the substantial increase in sedimentation documented in 2010 and 2011 in these cores, the benthic foraminiferal density alone did not account for compaction or dilution [9]. To account for compaction and dilution, a benthic foraminiferal accumulation rate (BFAR) approach was taken to determine changes from the down-core section to the upper section of each record [39,40]. BFAR were reported as the number of foraminifera per unit area over time (fcm^−2^yr^−1^).

### Redox Metal Concentrations

Redox metal concentration methods and data can be found in Hastings et al. (2014).

### Organic Geochemistry

EPA methods (8270D, 8015C) [41,42] and QA/QC protocols were followed for the analysis of hydrocarbons. Freeze-dried samples were extracted under high temperature (100°C) and pressure (1500 psi) with a solvent mixture 9:1v:v dichloromethane: methanol (MeOH) using an Accelerated Solvent Extraction system (ASE 2000, Dionex). Two extraction blanks were included with each set of samples (15–20 samples). The aromatic fraction was separated using solid-phase extraction (SiO_2_/C_3_-CN, 1 g/0.5 g, 6 mL) and hexane/dichloromethylene (3:1, v:v) as the solvent. PAHs were quantified using a gas chromatograph/mass spectrometric detector (GC/MS) in full scan mode (*m/z* 50–550) and splitless injections of 1μL. Oven temperature was 60°C for 8 min, increased to 290°C at a rate of 6°C/min and held for 4 min, then increased to 340°C at a rate of 14°C/min, and held at the upper temperature for 5 min. Concentrations of PAHs were calculated using response factors by comparison with a known standard mixture (16-unsubstituited EPA priority and selected isomers: Ultrascientific US-106N PAH mix, NIST 1491a) and were corrected for the recovery of the surrogate standard (d_10_-acenaphthene, d_10_-phenanthrene, d_10_-fluoranthene, d_12_-benz(a)anthracene, d_12_-benzo(a)pyrene, d_14_-dibenz(ah)anthracene, d_14_-benzo(ai)perylene). Recoveries from spiked samples were generally within 60–120%.

Prior to analysis of TOC, pre-weighed subsamples were acidified (80% 1.0N HCl) to remove inorganic carbon. Dried subsamples were placed in silver capsules and analyzed using a CarloeErba 2500 Series 2 Elemental Analyzer coupled to a Thermo Finnigan Delta XL. All samples were analyzed in duplicate and data reported as the average (<1% difference between duplicates). Detailed methods can be found in Romero et al. 2014 [29].

## Results

Pre-DWH and post-DWH sections of each sedimentary record were established through short-lived radionuclide (^210^Pb and ^234^Th) geochronologies (Table 1)[9].

**Table 1:** Short-lived radioisotope (^210^Pb, ^234^Th) activities, constant rate of supply age model, total organic carbon (TOC) percentages and TOC accumulation rates with depth for each core [29].

**Depth**	**Excess**	**^210^Pb**	**Excess**	**^234^Th**	**CRS**	**CRS**	**TOC**	**TOC Acc.**
	^**210**^ **Pb**	**Error**	^**234**^ **Th**	**Error**	**Date**	**Error**		**Rate**
**(mm)**	**(dpmg** ^**−1**^)	**(1σ)**	**(dpmg** ^**−1**^)	**(1σ)**	**(year)**	**(1σ)**	**(%)**	**(gcm** ^**−2**^ **yr** ^**−1**^)
**December 2010 DSH08**								
2	71.8	2.1	10.2	1	2010.9	1.3	2	135
4	71.8	1.7	9.1	0.8	2010.9	1.3	1.9	131
6	69.9	1.6	6.7	0.7	2010.8	1.3	2	136
8	70.3	1.4	5.6	0.6	2010.8	1.3	2	135
10	69.7	1.3	5.1	0.5	2010.7	1.3	2	
12	61.4	1.8	4.3	0.8	2009.6	1.3	2	**Pre-2010**
14	56.5	1.1	4.6	0.5	2008.5	1.3	2	11.4–12.6
16	63.3	1.2	4.2	0.5	2007.5	1.3	2	
18	51.6	1.1	3.8	0.5	2006.5	1.3	2	
20	51.7	1.1	3.9	0.5	2005.6	1.3	1.9	
30	44.3	1	4.3	0.2	2000.7	1.4	2	
35	38.3	0.9	3.9	0.4	1997.2	1.4	1.8	
40	41.6	0.9	4.1	0.4	1996	1.4	1.9	
45	39.1	0.9	3.7	0.4	1993.1	1.4	1.9	
50	35.2	0.8	3.8	0.4	1990.1	1.5	1.8	
55	38.8	0.9	3.3	0.4	1987	1.5	1.8	
60	32.6	0.8	3.6	0.4	1985.3	1.5	1.8	
70	26.7	0.4	2.7	0.2	1983.3	1.6		
80	17.3	0.4	2.4	0.2	1965.8	2		
90	10.3	0.3	2.7	0.2	1945.9	2.8		
110	5.4	0.2	3.1	0.2	1923.3	4.2		
130	2.2	0.2	3.3	0.2	1899.1	6.4		
140	1.7	0.2	2.9	0.2	1888.5	7.3		
150	1	0.3	2.5	0.3				
160	1.1	0.2	3.1	0.2				
**February 2011 DSH08**								
2	71.3	2.3	11.3	0.6	2011.1	1.6	1.9	61.9
4	62.8	1.8	6.6	0.3	2011	1.6	1.6	7.4
6	65.8	2.1	7.1	0.4	2010.9	1.6	2	135
8	59.9	1.8	5.9	0.3	2010.4	1.6	1.9	131
14	57.4	1.6	5.5	0.3	2008.9	1.7	2	**Pre-2010**
18	63.2	1.7	6.3	0.3	2007.1	1.7	2	11.4–12.6
24	57.2	1.6	4.7	0.2	2005.2	1.7		
30	51.3	0.3	4.7	0	2003	1.8	1.9	
34	50.7	1.2	4.1	0.2	2000.4	1.8	1.9	
36	47.3	1.3	4.3	0.2	1997.4	1.8		
40	42.9	1.1	4.2	0.2	1994	1.9	1.8	
50	22.2	0.6	4	0.2	1990.5	1.9	1.9	
70	20	0.5	2.4	0.1	1986.3	2.1	1.9	
90	13	0.5	2	0.1	1981.5	2.3		
110	9.3	0.5	2	0.1	1975.6	2.8	1.8	
130	5.9	0.4	2.1	0.1	1966.9	3.6	1.8	
150	3.3	0.5	2.1	0.1	1954.5	4.9	1.8	
170	1.3	0.3	2.2	0.1	1939.8	7.3		
180	0.8	0.5	1.9	0.1	1922.9	11.8		
200	0	0.4	2	0.1	1902.2	19.8		
**December 2010 PCB06**								
2	67.6	2.3	11.6	1.2	2010.9	1.6	1.3	95.8
4	66.9	1.5	5	0.6	2010.9	1.6	1.2	90.1
6	67.1	1.3	5.7	0.6	2010.7	1.6	1.2	6.4
10	57.5	0.8	2.6	0.3	2009.3	1.7	1.2	**Pre-2010**
12	60.2	1.1	3.5	0.4	2005.9	1.7	1.2	6.3–8.6
14	55.6	1.1	3.4	0.5	2003.8	1.7	1.2	
16	56	1.1	2.7	0.5	2002	1.8	1.2	
18	49.1	1	3.9	0.5	2000.1	1.8	1.3	
20	46.1	0.9	3.5	0.4	1998.3	1.8	1.3	
30	34.4	0.8	4.1	0.4	1990.7	2	1.4	
38	30.2	0.8	3.1	0.4	1983.3	2.2	1.5	
42	28.4	0.8	3.7	0.4	1979.9	2.3	1.5	
52	20.3	0.8	3.5	0.4	1970.5	2.6	1.4	
62	15.8	0.5	2.8	0.3	1962.1	2.9	1.3	
72	12.3	0.6	3.1	0.3	1951.8	3.4	1.3	
82	10	0.7	3.9	0.4	1940.8	4	1.4	
92	6.9	0.5	3.3	0.4	1929	4.7	1.5	
105	2.2	0.3	1.8	0.2	1920.2	5.2	1.4	
115	2.3	0.3	1.8	0.2	1915.3	5.4	1.7	
135	2	0.2	2.1	0.2	1902.9	6.2	1.5	
155	1.3	0.2	2	0.2	1887.2	7.4		
**February 2011 PCB06**								
2	69.2	1.2	9.6	0.5	2011.1	1	1.9	12.1
6	55.3	1	4.6	0.3	2009.8	1	1.3	95.8
10	51.1	1	5.4	0.3	2008.3	1	1.2	90.1
14	54.9	0.9	4.1	0.2	2006.8	1	1.2	6.9
18	78.4	1.1	3.5	0.2	2004.7	1	1.2	**Pre-2010**
26	65.2	1	3.4	0.2	1999.2	1	1.2	6.3–8.6
34	63.8	1	4.4	0.3	1993.5	1.1	1.2	
50	47.8	0.9	4	0.2	1978.9	1.2	1.2	
66	30.2	0.7	4	0.2	1960.8	1.4	1.3	
75	15.9	0.3	2.5	0.1	1954.1	1.6	1.3	
95	6.6	0.2	2.3	0.1	1936.7	2	1.4	
125	3.3	0.2	2.8	0.1	1912.8	2.6	1.5	
155	2	0.2	3	0.1	1885	3.5	1.5	

### Benthic foraminiferal density with depth

**Fig 2:**
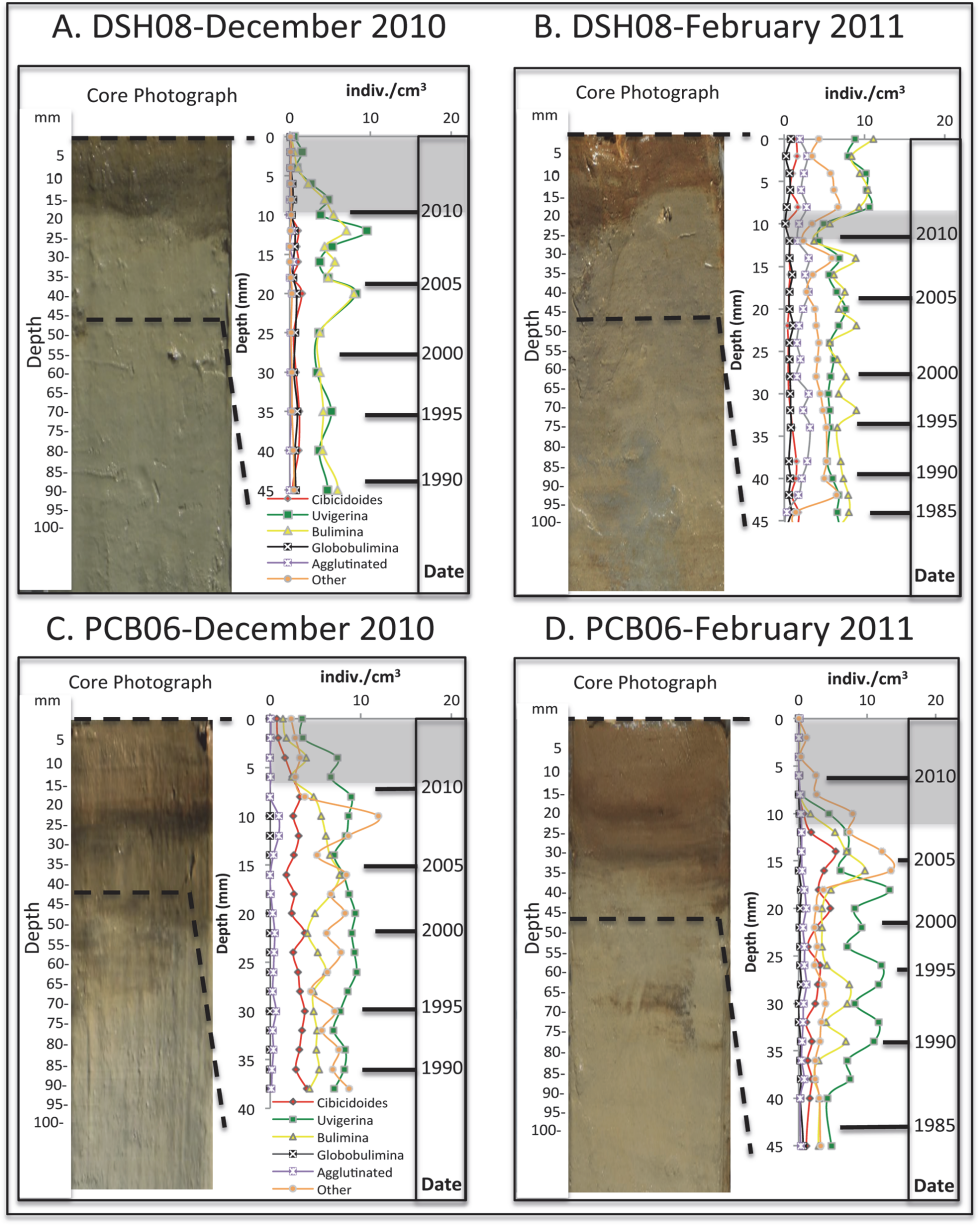
Core photographs and densities (indiv./cm^3^) of the benthic foraminifera genera throughout the surface sections of each core site including gray areas representing the decline in benthic foraminifera density in each record and the corresponding date.

The dominant genera throughout the December 2010 and the February 2011 records from DSH08 were *Bulimina* spp. and *Uvigerina* spp. (Fig. 2 A-B). The mean density in the December 2010 and February 2011 records of *Bulimina* spp. (4.1 and 6.8 indiv./cm^3^, respectively) and *Uvigerina* spp. (4.0 and 6.1 indiv./cm^3^, respectively) were much higher than any of the other genera. In the December 2010 record, the relative abundance of each genus remained the same throughout the lower section from 25–45 mm (pre-DWH). In the surface section (0–12 mm, post-DWH) of the core, there was a sharp decrease in all of the genera with the most pronounced decrease in *Bulimina* spp., *Uvigerina* spp. and *Cibicidoides* spp. *Bulimina* spp. density decreased from 5.4 indiv./cm^3^ at 10 mm to 0.2 indiv./cm^3^ at the surface, *Uvigerina* spp. density decreased from 3.8 indiv./cm^3^ at 10 mm to 0.5 indiv./cm^3^ at the surface, and *Cibicidoides* spp. decreased from 0.14 indiv./cm^3^ at 10 mm to 0.07 indiv./cm^3^ at the surface. The down-core (pre-DWH) section of the February 2011 record (15–45 mm) resembled the December 2010 record with very little variation in the relative abundance of each genus. The most apparent trend in the February 2011 record from DSH08 was the decrease in all genera densities at 10–12 mm (2010 CE), especially *Bulimina* spp. (3.7 indiv./cm^3^) and *Uvigerina* spp. (4.3 indiv./cm^3^), which was similar to the decrease in the surface (post-DWH) of the December 2010 record. This noticeable decrease was followed by an increase towards the surface (0–8 mm) to densities greater than the down-core record (15–45 mm). The surface (0–8 mm, post-DWH) was very similar to the down-core (15–45 mm, pre-DWH) section except for the increase in *Bolivina* spp. (“other” category) relative to the dominant *Bulimina* spp. and *Uvigerina* spp.

In contrast to the DSH08 records, the dominant genus throughout the entire December 2010 and February 2011 PCB06 records was *Uvigerina* spp. (mean-7.7 and 6.1 indiv./cm^3^, respectively) (Fig. 2 C-D). With the exception of increases in *Brizalina* spp. (“other”) at 10 mm (11.9 indiv./cm^3^) and in *Bulimina* spp. (7.6 indiv./cm^3^) at 16 mm, there was very little variation in the density of each genus up-core from 38 mm to 8 mm (pre-DWH) in the December 2010 record. From 8 mm to the surface of the record (post-DWH), there was an overall decrease in all of the genera (e.g. *Uvigerina* spp. 8.9 indiv./cm^3^ at 8 mm and 3.5 indiv./cm^3^ at the surface). This decrease was also evident and continued in the February 2011 record from 15 mm to the surface (post-DWH), where the density of every genus, with the exception of *Cibicidoides* spp. decreased to zero.

*Uvigerina* spp. was the dominant genus from 35 to 10 mm in the NT1200 record (Table 2). In the surface section of the core (0–10 mm), *Uvigerina* spp., *Cibicidoides* spp., and *Bulimina* spp. all increased in density. Despite these relative increases in density in the surface section of this core, the total density only increases slightly from the down-core section (25.2 indiv./cm^3^, 10–50 mm) to the surface section (28.9 indiv./cm^3^, 0–10 mm).

**Table 2:** Number of dominant genera with depth for each core and the corresponding BFAR.

**Depth**	***Cibicidoides***	***Uvigerina***	***Bulimina***	***Globobulimina***	**Agglut.**	**Other**	**Total**	**BFAR**
**(mm)**	**spp. (#)**	**spp. (#)**	**spp. (#)**	**spp. (#)**	**(#)**	**(#)**	**(#)**	**(fcm** ^−**2**^ **yr** ^−**1**^)
**December 2010 DSH08**								
2	1	7	3	1	3	2	17	1.9
4	3	21	8	1	1	2	36	1
6	0	12	14	2	2	2	32	0.8
8	1	37	32	5	2	2	79	2.2
10	5	67	60	6	0	3	141	2.4
12	2	53	76	4	0	3	138	4.5
14	15	134	98	9	4	1	261	3.7
16	12	73	61	9	0	0	155	2.5
18	15	52	78	6	6	0	157	3.1
20	6	66	66	6	0	2	146	3.1
25	21	115	111	12	4	4	267	
30	18	130	133	26	4	6	317	1.7
35	28	122	134	19	5	10	318	2.1
40	42	185	150	35	7	11	430	2.3
45	40	132	146	24	0	17	359	2.3
50	14	169	214	27	1	19	444	2.5
55	46	126	115	32	1	12	332	1.8
60	26	129	113	29	1	12	310	2.7
70	23	123	133	33	2	14	328	1.6
75	18	132	172	30	5	24	381	
80	32	133	172	20	2	30	389	1.8
85	32	147	153	22	3	15	372	
90	25	131	190	18	1	22	387	1.4
**February 2011 DSH08**								
2	21	123	156	12	27	60	399	3.8
4	22	111	117	3	40	48	341	6.9
6	14	143	132	9	34	81	413	5.8
8	11	144	146	10	30	87	428	5
10	24	148	131	5	39	94	441	4.7
12	4	69	79	2	26	48	228	3.2
14	9	60	52	9	24	33	187	2.1
16	9	96	125	9	43	83	365	
18	10	78	86	14	37	50	275	2.6
20	8	91	106	9	43	39	296	
22	8	107	95	9	33	53	305	
24	6	95	126	15	26	56	324	
26	7	80	79	9	22	60	257	2.4
28	7	86	92	9	28	58	280	
30	7	81	108	10	22	56	284	3.1
32	8	77	95	11	42	63	296	
34	11	79	126	11	33	68	328	2.5
36	14	79	93	12	45	73	316	
40	21	75	98	8	40	74	316	
42	20	84	103	11	31	70	319	2
44	13	95	112	8	25	91	344	
46	25	92	113	12	4	20	266	
48	24	94	94	4	3	21	240	
50	29	68	81	8	20	61	267	1.5
55	51	192	143	19	43	123	571	
60	46	156	168	17	28	61	476	
65	64	157	181	19	61	103	585	
70	38	125	126	13	96	151	549	
75	51	189	213	25	72	108	658	1.8
80	38	161	176	22	46	88	531	
85	45	139	190	17	28	43	462	
90	32	171	181	13	4	82	483	
95	41	178	184	27	5	75	510	1.2
100	40	214	201	24	1	112	592	
**December 2010 PCB06**								
2	10	49	20	n.d.	1	32	112	5.1
4	12	50	25	n.d.	1	39	127	6.1
6	23	104	56	n.d.	0	46	229	8.2
8	34	93	33	n.d.	1	39	200	8.9
10	46	125	67	n.d.	0	53	291	12.7
12	36	121	79	n.d.	13	167	416	12.6
14	44	117	86	n.d.	13	121	381	10.8
16	37	99	92	n.d.	5	72	305	8.2
18	25	113	107	n.d.	0	118	363	9.7
20	37	122	94	n.d.	1	93	347	8.4
22	33	131	69	n.d.	4	115	352	8.4
24	53	126	58	n.d.	7	87	331	6.3
26	36	130	73	n.d.	6	109	354	8
28	43	133	86	n.d.	5	88	355	10.8
30	46	120	67	n.d.	4	63	300	7.5
32	53	108	67	n.d.	9	100	337	5.8
34	49	98	72	n.d.	4	80	303	5.8
36	45	116	71	n.d.	5	106	343	6.1
38	40	114	75	n.d.	3	97	329	6.7
40	57	99	61	n.d.	2	122	341	7.3
**February 2011 PCB06**								
0	2	0	0	0	0	2	4	0.2
2	0	1	2	1	2	1	7	0.5
4	0	1	2	0	0	15	18	1.6
6	0	0	1	0	0	4	5	0.3
8	1	2	1	0	1	35	40	1.7
10	1	6	2	0	3	37	49	1.8
12	10	62	24	2	5	111	214	6.9
14	25	99	74	4	6	104	312	10.3
16	75	100	98	5	6	170	454	11.7
18	52	86	135	1	7	189	470	7.5
20	41	186	66	5	12	50	360	6.2
22	64	115	48	3	15	36	281	4.5
24	36	129	48	2	9	32	256	4.3
26	19	99	48	3	12	38	219	3.3
28	43	169	58	3	12	33	318	4.7
30	40	163	103	5	16	50	377	4.8
32	34	115	99	0	10	55	313	3
34	15	163	56	1	9	46	290	3.3
36	27	154	97	4	7	43	332	3.4
38	18	99	41	3	6	34	201	2.3
40	27	105	32	5	11	34	214	n.d.
45	58	151	109	8	8	109	443	n.d.
50	41	172	104	23	10	115	465	n.d.

Areas where there are no data available are denoted “n.d.”.

### Benthic foraminiferal accumulation rates (BFAR)

**Fig 3:**
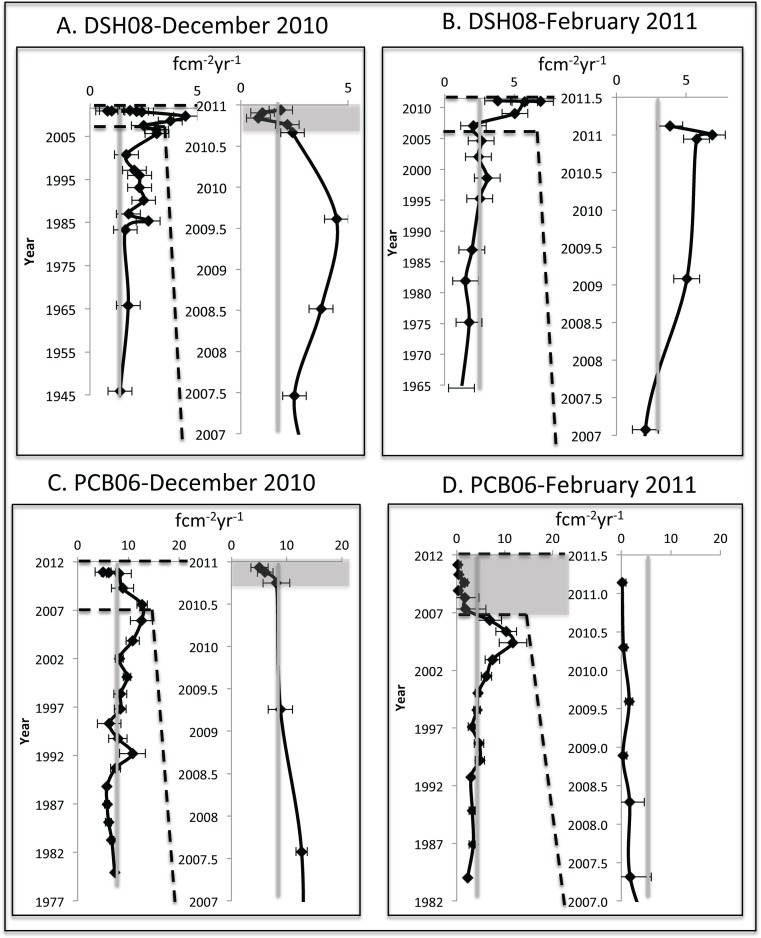
Benthic foraminiferal accumulation rate (BFAR) records for each sampling site with the period from 2007–2011 expanded for examination of the period during and after the DWH event. The gray areas represent the decline in BFAR and the gray lines represent the down-core mean BFAR.

The benthic foraminiferal accumulation rate (BFAR) record from the DSH08 core collected in December 2010 ranged from 1.0–4.5 fcm^−2^yr^−1^ (Fig. 3A, Table 2). From 1945 (1.4 fcm^−2^yr^−1^) to 2000 (1.7 fcm^−2^yr^−1^) the BFAR remained relatively constant. There was an increase from 2005 (3.1 fcm^−2^yr^−1^) to 2009 (4.5 fcm^−2^yr^−1^). In late 2010, the BFAR (1.0 fcm^−2^yr^−1^) decreased below the rate in the bottom section of the core (1945–2000 CE).

The BFAR from the DSH08 record collected in February 2011 ranged from 1.2 fcm^−2^yr^−1^ to 6.9 fcm^−2^yr^−1^ (Fig. 3B). There was relatively little variation in BFAR from 1965 (1.2 fcm^−2^yr^−1^) to 2007 (2.1 fcm^−2^yr^−1^). The BFAR then increased from 2009 (5.0 fcm^−2^yr^-1^) to 2011, where the mean BFAR for the two surface samples was 5.4 fcm^−2^yr^−1^.

The BFAR record from the PCB06 site in December 2010 ranged from 5.1 fcm^−2^yr^−1^ to 12.7 fcm^−2^yr^−1^ (Fig. 3C). During late 2010, there was a decrease from 8.5 fcm^−2^yr^−1^ to 5.1 fcm^−2^yr^−1^ at the surface.

The PCB06 BFAR record from February 2011 ranged from 0.2–11.7 fcm^−2^yr^−1^ (Fig. 3D). There was a gradual increase in BFAR (2.2–11.7 fcm^−2^yr^−1^) throughout the bottom section of the record (1984–2006). This was followed by a gradual decrease from 2007 (1.7 fcm^−2^yr^−1^) to early 2011 (0.2 fcm^−2^yr^−1^). The PCB06 dating and accumulation rate records from February 2011 were not coupled with ^234^Th, and were purely based on ^210^Pb, which may not have resolved the flocculent pulse in the surface portion of this core and could have affected the dates in the surface 15 mm.

### Environmental controls on foraminiferal densities

**Fig 4:**
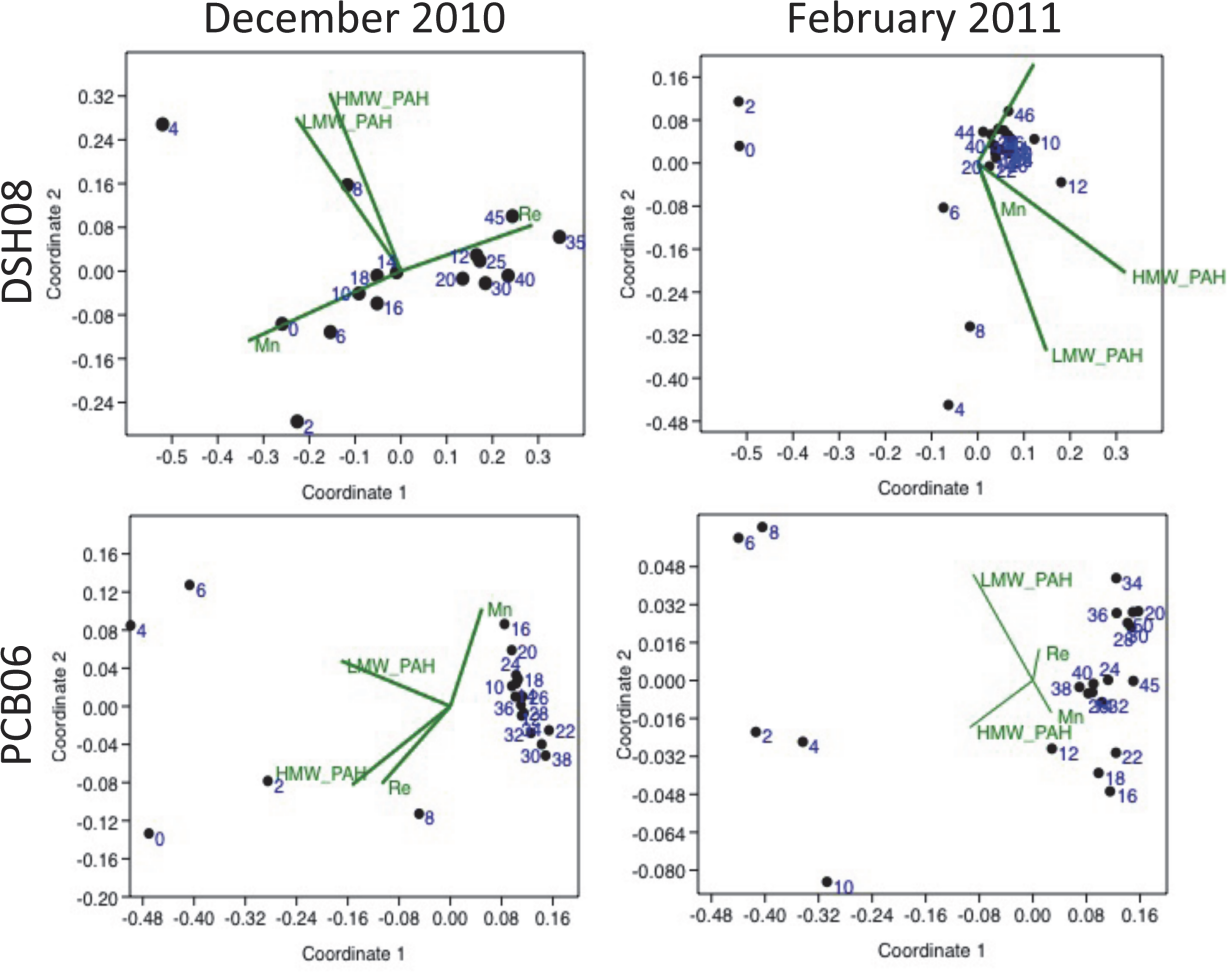
Non-metric multidimensional scaling plots for each core, where black dots represent the foraminiferal density at each sample interval (depths labeled in blue) and green vectors represent each environmental variable (HMW PAH, LMW PAH, Re, Mn)[28,29]. The Euclidian distance between each sample depth represents the Bray Curtis similarity and the orientation and length of the green vectors represent the correspondence and intensity of each environmental parameter to the variability between foraminiferal density.

Non-metric multidimensional scaling (nMDS) plots were utilized to distinguish the similarities between foraminiferal densities in each sample increment and the corresponding environmental variable (LMW PAH, HMW PAH, Re, Mn)(Fig. 4). The most notable trend in every core was the separation of the surface interval denisites (∼0–10 mm) from the down-core interval densities (10–50 mm). The separation of the surface interval from the down-core interval in every record was driven by PAH concentration (both HMW and LMW), whereas any variability in foraminiferal density below 10 mm was driven by redox processes (Re,Mn).

## Discussion

### General comparison with previous records

*Uvigerina* spp., *Bulimina* spp., and *Cibicidoides* spp. were the dominant genera in the down-core section of PCB06 and DSH08, in upper and lower Gulf of Mexico slope sediments found by Osterman [26], as well as the outer shelf and slope assemblages described by Culver and Buzas [23]. There were also similarities between the CMW assemblage [24] from 850–1500m water depth, and the down-core (below 12 mm, pre-DWH) records from DSH08, which were both dominated by *Bulimina* spp. and secondarily by *Cibicidoides* spp. The post-DWH interval from the February 2011 DSH08 record (0–8 mm) was also similar to the CMW assemblage described in Denne and Sen Gupta [24] with the exception of an increase in *Bolivina* spp. While agglutinated genera were present in every sample at each site, they were certainly not the dominant genera at either of the sampling sites. This disagreement with Bernhard et al. [27] might have been due to the loss of some agglutinated foraminifera in the freeze-drying and wet sieving methods. There were not enough benthic foraminifera in the 10–12 mm (DWH event, 2010 CE) section of the February 2011 DSH08 record to compare with previous studies. The down-core (15–40 mm) PCB06 intervals (pre-DWH) shared two of the dominant species (*Bolivina* spp. and *Uvigerina* spp.) with the hydrocarbon seep communities presented in Sen Gupta and Aharon [25]. However, these records differed with respect to the dominance of *Bulimina* spp. in PCB06 and lack of *Bulimina* spp. in most of their hydrocarbon seep sites (F1, F12, F15). There were not enough benthic foraminifera, epifaunal or infaunal, in the surface (post-DWH) of the PCB06 cores to compare dominant genera with previous records.

### Decline in benthic foraminiferal density

**Fig 5:**
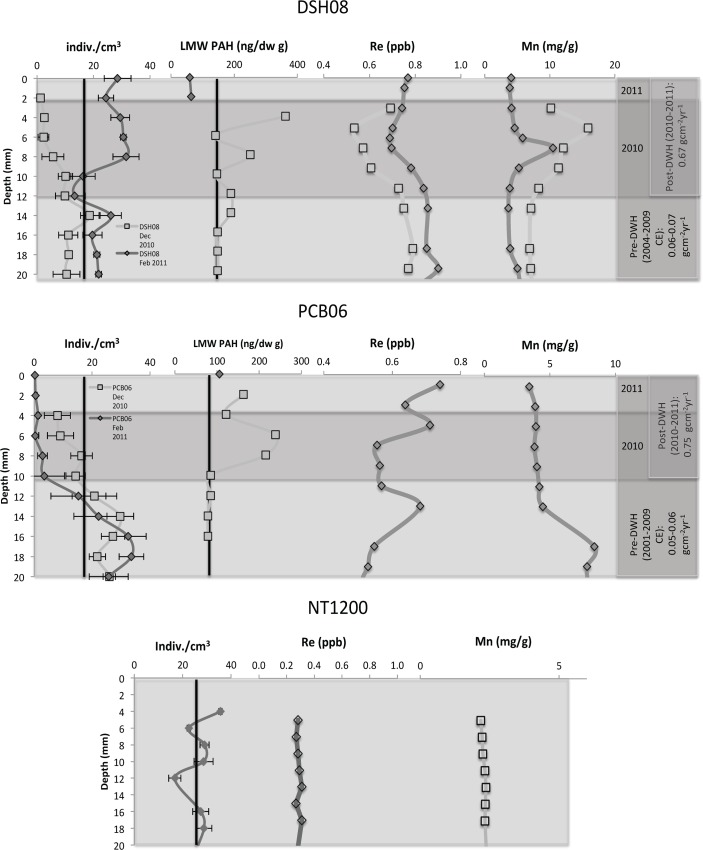
Total benthic foraminiferal density (indiv./cm^3^), low molecular weight polycyclic aromatic hydrocarbon concentrations (LMW PAH, ng/g dw)[29], and redox sensitive metal (Re, Mn)[28] records stacked using short-lived radioisotope geochronology. Pre-DWH and post-DWH periods are denoted with their respective mass accumulation rates in the gray-shaded areas and the down-core means for benthic foraminiferal density and LMW PAH are represented by black lines.

There was no decline in benthic foraminiferal density at the NT1200 control site (Fig. 5, Table 2). However, a decline (i.e. a continuous decrease below down-core mean) was evident in benthic foraminiferal density (all genera, infaunal and epifaunal) and BFAR in the surficial 10 mm at the PCB06 and DSH08 sites in December 2010. The records from February 2011 suggested that a possible recovery from the decline was site specific. At the DSH08 site, there was evidence of the decline from 10–12 mm (2010 CE) and a subsequent increase (apparent recovery) in density and BFAR records in the surface section (0–10 mm, post DWH)). At the PCB06 site, the decline in the surface section (0–8 mm) of the December 2010 record was continued in the 2011 record, where the density reached near-zero values (four individuals) in the surface sample of the core (post-DWH), compared to several hundred (264 individuals) from 10–12mm (pre-DWH). In the PCB06 record from February 2011, it appeared that the decline begins prior to 2010 in the geochronological record. Due to the lack of ^234^Th dating on this core, the discrepancy may have been due to the inability of the ^210^Pb geochronology to resolve the flocculent pulse at the surface.

Sen Gupta and Aharon [25] documented from 84–108 individuals and a total benthic foraminiferal density of 3–5.7 indiv./cm^3^ from core top sediments impacted by natural hydrocarbon seeps in the northern Gulf of Mexico that ranged from 500–700 m water depth (Table 3). Studies conducted previous to the DWH event reported much higher total benthic foraminiferal density (1000–3000 individuals, 13.1–29.1 indiv./cm^3^) from sites along the continental slope and rise (900–1850 m water depth), that were not associated with natural seeps [26,33]. The down-core mean from the four cores in this study was 19.6 indiv./cm^3^ and the mean density at the control site (NT1200) was 27.1 indiv./cm^3^. Both of these records were similar to the high density found by Osterman et al. and Rowe and Kennicutt [26,33] and therefore validate their representation of background and control values. The post-DWH mean for the four cores in this study was 7.9 indiv./cm^3^, similar to the low density found by Sen Gupta and Aharon [25] near the natural hydrocarbon seeps. The similarities between the density in the down-core (pre-DWH) sections of the DSH08 and PCB06 records compared with those from Osterman et al. and Rowe and Kennicutt [26,33] along with the similarities between the density in the post-DWH sections of the DSH08 and PCB06 records compared with those from Sen Gupta and Aharon [25] suggest that the sedimentary environment changed dramatically in the surface section of the DSH08 and PCB06 records.

**Table 3:** Mean impacted and control benthic foraminiferal densities (indiv./cm^−^^3^) from this study are presented with three previous records of total surface benthic foraminiferal densities (indiv./cm^−^^3^) collected at natural seep sites and along the continental slope of the northern Gulf of Mexico.

**Record**	**Sample Description**	**Water depth (m)**	**Impacted Density (indiv./cm^3^)**	**Control Density (indiv./cm^3^)**
**This Study**	DSH08 mean density:	1143	8.85	16.5
	impacted (0–10mm),			
	control (10–45mm)			
	PCB06 mean density:	1043	6.7	22.7
	impacted (0–10mm),			
	control (10–45mm)			
	NT1200 (control site)	1200	no impact	27.1
	mean density (0–50mm)			
**Sen Gupta and Aharon (1994)**	core tops impacted from	500–700	4.35	n.d.
	natural seeps (1992)			
**Osterman**	Unimpacted core tops from continental slope (2000)	900–1000	no impact	13.1
**−2002**				
**Rowe and Kenicutt (2009)**	Unimpacted core top from	1850	no impact	29.1
	DeSoto Canyon site S36 (2000)			

Given the lack of replication at each site and time-stamp, it is necessary to address the possibility that spatial patchiness could have been a factor in the variance in foraminiferal density between each site and time-stamp [43]. It was evident that the densities and relative abundances of the dominant genera were different between sites. This was expected considering the distance between the sites, the different sedimentary settings, and the difference in water depth. However, there was independent continuity at each site between the records collected in December 2010 and those in February 2011. For example, the relative abundance of the dominant genera in the December 2010 cores were very similar to those found in the February 2011 cores at both sites, especially in the down-core (pre-DWH) sections. There was also continuity in both the values and covariance in the total density records from December 2010 to those collected in February 2011 at both sites (Fig. 5). Considering the continuity, in not only the relative abundance of the dominant genera from one time-stamp to the next, but also the similarity in the total density values and covariance of the total density records, it was unlikely that patchiness caused any significant variations in the records from each time-stamp at each site.

### Evidence for sudden change in sedimentary environment

Brooks et al. [9] documented a widespread sedimentary pulse in late 2010 throughout the Northeastern Gulf of Mexico that produced a layer from 0.4–1.2 cm that was deposited in 4–5 months. The continuous decay of ^234^Th and ^210^Pb activity with depth and the lack of step-wise alterations down-core indicated a lack of bioturbation or vertical mixing throughout each record (Table 1). Brooks et al. [9] provide comprehensive sedimentological evidence of lamination and the lack of vertical mixing in the surface intervals (0–10 mm) of these core records. At the DSH08 site, the total sedimentary mass accumulation rate (MAR) increased from 0.06 gcm^−2^yr^−1^ (pre-DWH, 1903–2006 CE) to 0.67 gcm^−2^yr^−1^ (post-DWH, 2010–2011 CE) (Fig. 5). At the PCB06 site, the MAR increased from 0.06 gcm^−2^yr^−1^ (pre-DWH, 1902–2003 CE) to 0.75 gcm^−2^yr^−1^ (post-DWH, 2010–2011 CE). The result of this pulse was a finely laminated layer in the surface section (∼0–10 mm) at both PCB06 and DSH08.

An increased flux of organic carbon to the sediments would be expected to decrease the sedimentary pore-water oxygen concentration as the organic matter is decomposed. Altenbach et al. [44] presented a “high flux” North Atlantic benthic foraminifera assemblage that had a POC deposition rate between 2 x 10^–4^ gcm^−2^yr^−1^ and 2 x 10^–3^ gcm^−2^yr^−1^. Rowe et al., [45] measured a deposition rate of particulate organic carbon (POC) of approximately 5.7 x 10^–4^ gcm^−2^yr^−1^ for the mid-slope of the Gulf of Mexico. POC constituted about 2% of the MAR at the PBC06 and DSH08 sites [9,29]. With a MAR of ∼0.67 gcm^−2^yr^−1^ during 2010, the POC deposition rate was 1.3 x 10^–2^ gcm^−2^yr^−1^ (Table 1)[9,29]. In 2010, both sites experienced a high flux of POC associated with the flocculent blizzard.

Hastings et al. [28] determined changes in the redox state of sediments at DSH08 and PCB06 in December 2010 and February 2011 based on concentrations of several redox sensitive metals (Mn and Re)(Fig. 5). The DSH08 redox state records corresponded with the decrease in *Bulimina* spp. and *Uvigerina* spp. density at the surface in December (enriched Re) and at ∼12 mm in February (Fig. 5). These decreases in benthic foramiiferal density occurred at the same depth as Mn minima and Re enrichment (0.16 ppb), which indicated reducing conditions [28]. Similar corroboration was found in the PCB06 records. At the surface of PCB06, in both the December 2010 and February 2011 records, there was a significant decrease in the density of benthic foraminifera that corresponded to the Mn minimum and an increase in Re concentration (Dec. 0.07 ppb, Feb. 0.18 ppb Re increase). Hastings et al., [28] also found reducing sediments throughout the surface section of the February 2011 PCB06 record, which suggested that reducing (sub-oxic) conditions persisted at this site for as many as ten months. There was no evidence of reducing sediments in the surface 50 mm at the NT1200 control site (Fig. 5).

Oil droplet models suggested that the subsurface intrusion from 1000–1300 m water depth impinged on the continental slope near the two sites discussed in this study (PCB06 and DSH08) [7]. The droplet and dissolved portions of the intrusion included PAHs [1, 42]. These compounds were likely also present in the pulse of flocculent material that was deposited in late 2010 [3,8]. During the Deepwater Horizon event, the sedimentary low molecular weight PAH concentration increased in 2010 from ∼100 ng/g(OC) (background) to higher than 200 ng/g(OC) at PCB06 and from ∼200 ng/g(OC) (background) to higher than 350 ng/g(OC) at DSH08 (Fig. 5)[29].

### Potential mechanisms for benthic foraminiferal decline

Possible mechanisms that may have caused the persistent decline in benthic foraminiferal density in the surface of the PCB06 cores are: (1) increased predation, (2) lateral or vertical foraminifera movement, (3) mortality, (4) inhibition of reproduction, and (5) dilution. The fact that the sedimentological and radioisotope (^234^Th) records [9] showed a lack of bioturbation in the surface section (laminations from ∼0–10 mm) of these cores, eliminates predation and lateral or vertical movement. Increased predation from detritivorous meiofauna or macrofauna would have produced an increased record of bioturbation throughout the surface section of the core. Considering that the decline affected every genus, vertical or lateral movement of the foraminifera would also have increased bioturbation in the ^234^Th and sedimentological record [46–49]. Also, considering the widespread and sudden nature of the sedimentation event, it is unlikely that lateral movement would account for a decline in surface density on this scale (m to km) due to the relatively slow movement of foraminifera [46,47]. As previously stated, dilution has been ruled out by using benthic foraminiferal accumulation rates. The only two remaining mechanisms are mortality and inhibition of reproduction, which our methods alone cannot directly constrain (lack of staining).

It has been demonstrated that many foraminifera genera can survive anoxic conditions [50–55]. Risgaard-Petersen et al., [51] documented cases of *Globobulimina pseudospinescens* surviving for over a month in anoxic conditions by denitrifying stores of nitrate. Piña-Ochoa et al., [52] described several genera (*Bulimina* spp., *Uvigerina* spp., and *Bolivina* spp.) as facultative anaerobes, where cell maintenance and food gathering was possible under anoxic conditions. However, Piña-Ochoa et al., [52] found that oxygen respiration rates were much higher (3–13 times) than denitrification rates, which suggests that oxygen may be necessary for reproduction and growth. Furthermore, the evidence that foraminifera in anoxic conditions for long periods of time (weeks to months) must migrate vertically (not simply extend pseudopodia) to access nitrate [52], along with the lack of bioturbation in the surface of these cores [9], suggests an increase in mortality. Langlet et al. [55] found that a significant portion (∼25–30%) of the original living foraminifera (oxic) could survive up to ten months in anoxic conditions. However, prolonged anoxia caused a decline in the original density by ∼70–75%, which suggested that prolonged anoxia could cause a significant decrease in benthic foraminiferal density [55]. The fact that the decline in density and records of reducing conditions [28] persisted in the surface section (∼10 mm) of the February 2011 record (10 months after the DWH event), it is possible that reducing conditions contributed to the decline by inhibiting reproduction or causing mortality.

Montagna et al. [59] documented severe reduction in abundance of all benthic fauna related to DWH impacts. Benthic foraminiferal exposure to PAH’s has been shown to increase mortality rates and decrease reproduction [15,56,57]. Prolonged exposure (weeks) of benthic foraminifera to PAHs at high concentrations (HMW PAH- 4.9 mg/g, LMW PAH-0.1 mg/g) has been related to cases of complete mortality [56]. The PAH concentrations in the surficial interval (0–10 mm) at DSH08 (145–362 ng/g) and PCB06 (131–238 ng/g) in December 2010 and February 2011 were well below the concentrations reported by Ernst et al. (2006)[29,56]. However, the PAH concentrations in the surface interval (0–10 mm) still increased 2–3 fold relative to baseline (down-core) concentrations and increased PAH concentrations occurred at the same depth as the decline in benthic foraminifera for as many as ten months after the DWH event. Mojtahid et al. (2006) [17] reported declines in density and dominance of opportunistic taxa such as *Bulimina spp*. and *Bolivina spp*. at drill cutting disposal sites with total petroleum hydrocarbon (TPH) concentrations ranging from 16–111 mg/g. The baseline (down-core) TPH concentrations at PCB06 and DSH08 in December 2010 and February 2011 ranged from 0.5–1.3 mg/g and increased to 17 mg/g in the surface interval (0–10 mm) [29]. The nMDS results also suggested that PAH concentrations were the dominant driver of variability in foraminiferal density in the surface interval (0–10 mm) of every core (Fig. 4). Considering the documented toxicity of PAHs [15], their effects on reproduction in benthic foraminifera [52] and the sudden nature of the DWH event [9], it is possible that the benthic foraminifera at DSH08 and PCB06 were either not able to adapt quickly enough to such a significant increase in PAHs or could not withstand their persistent toxicity [15,56].

## Conclusions

1. The benthic foraminifera in the down-core (pre-DWH) intervals (>10 mm) at DSH08 and PCB06 resembled assemblages from previous GoM studies related to Caribbean Midwater mass [24] and continental slope sediments [23,26].
2. An 80–93% decline in the density of all genera (infaunal and epifaunal) of benthic foraminifera has been documented in the surface section (∼0–10 mm) at both of the impacted sampling sites in December 2010 in contrast to the control site.
3. The decline in the density of benthic foraminifera occurred simultaneously with abrupt and widespread increases in sedimentary accumulation rates, PAH concentrations, and changes in redox conditions.
4. The records from February 2011 suggested a site-specific response:
  -There was evidence of a decline and a possible, subsequent recovery in the density and accumulation rate of benthic foraminifera at the DSH08 site. Cores collected after February 2011 will provide further evidence of the potential recovery.
  -There was evidence of a continued decline with near-zero values at the surface of the February 2011 PCB06 record. Again, cores collected after February 2011 will provide evidence of the longevity of the decline.
5. Persistent reducing conditions (10 months after DWH event) in the surface of these cores were a possible contributor to the continued decline at PCB06 due to mortality or inhibited reproduction.
6. Although our methods could not directly constrain a mortality event (lack of staining), it was likely that the decline in density in the surface of these core records was caused the synchronous, significant increase in concentration of low molecular weight PAHs attributed to the sudden and widespread nature of the DWH event seeing as these compounds are known to be toxic to foraminifera [15,56].

The paired analysis of benthic foraminiferal density records with short-lived geochronology, redox sensitive metal concentrations, and organic chemistry is a robust tool in assessing the impact of the Deepwater Horizon event. This analysis allows for a basic understanding of how deep-water petroleum emissions can affect the benthic habitat health. It identifies specific biological impacts related to the physical and chemical changes in the water column and sediments. It also provides an estimate for the time needed for the benthic communities to recover after a deep-water petroleum emission. By continuing to use this analytical approach it will be possible to also document the long-term recovery from and effects of the Deepwater Horizon event.
