# Correction: A Decline in Benthic Foraminifera following the Deepwater Horizon Event in the Northeastern Gulf of Mexico

**DOI:** 10.1371/journal.pone.0128505

**Published:** 2015-05-19

**Authors:** Patrick T. Schwing, Isabel C. Romero, Gregg R. Brooks, David W. Hastings, Rebekka A. Larson, David J. Hollander

Table 1 appears incorrectly in the published article. Please see the correct Table 1 here.

**Table 1:** Short-lived radioisotope (^210^Pb, ^234^Th) activities, constant rate of supply age model, total organic carbon (TOC) percentages and TOC accumulation rates with depth for each core [29].

Depth	Excess ^210^Pb	^210^Pb Error	Total ^234^Th	^234^Th Error	CRS Date	CRS Error	TOC	TOC Acc. Rate
(mm)	(dpmg^-1^)	(1σ)	(dpmg^-1^)	(1σ)	(year)	(1σ)	(%)	(gcm^-2^yr^-1^)
**December 2010 DSH08**								
2	71.8	2.1	10.2	1.0	2010.9	1.3	2.0	135.0
4	71.8	1.7	9.1	0.8	2010.9	1.3	1.9	131.0
6	69.9	1.6	6.7	0.7	2010.8	1.3	2.0	136.0
8	70.3	1.4	5.6	0.6	2010.8	1.3	2.0	135.0
10	69.7	1.3	5.1	0.5	2010.7	1.3	2.0	
12	61.4	1.8	4.3	0.8	2009.6	1.3	2.0	**Pre-2010**
14	56.5	1.1	4.6	0.5	2008.5	1.3	2.0	11.4–12.6
16	63.3	1.2	4.2	0.5	2007.5	1.3	2.0	
18	51.6	1.1	3.8	0.5	2006.5	1.3	2.0	
20	51.7	1.1	3.9	0.5	2005.6	1.3	1.9	
30	44.3	1.0	4.3	0.2	2000.7	1.4	2.0	
35	38.3	0.9	3.9	0.4	1997.2	1.4	1.8	
40	41.6	0.9	4.1	0.4	1996.0	1.4	1.9	
45	39.1	0.9	3.7	0.4	1993.1	1.4	1.9	
50	35.2	0.8	3.8	0.4	1990.1	1.5	1.8	
55	38.8	0.9	3.3	0.4	1987.0	1.5	1.8	
60	32.6	0.8	3.6	0.4	1985.3	1.5	1.8	
70	26.7	0.4	2.7	0.2	1983.3	1.6		
80	17.3	0.4	2.4	0.2	1965.8	2.0		
90	10.3	0.3	2.7	0.2	1945.9	2.8		
110	5.4	0.2	3.1	0.2	1923.3	4.2		
130	2.2	0.2	3.3	0.2	1899.1	6.4		
140	1.7	0.2	2.9	0.2	1888.5	7.3		
150	1.0	0.3	2.5	0.3				
160	1.1	0.2	3.1	0.2				
**February 2011 DSH08**								
2	71.3	2.3	11.3	0.6	2011.1	1.6	1.9	61.9
4	62.8	1.8	6.6	0.3	2011.0	1.6	1.6	7.4
6	65.8	2.1	7.1	0.4	2010.9	1.6	2.0	135.0
8	59.9	1.8	5.9	0.3	2010.4	1.6	1.9	131.0
14	57.4	1.6	5.5	0.3	2008.9	1.7	2.0	**Pre-2010**
18	63.2	1.7	6.3	0.3	2007.1	1.7	2.0	11.4–12.6
24	57.2	1.6	4.7	0.2	2005.2	1.7		
30	51.3	0.3	4.7	0.0	2003.0	1.8	1.9	
34	50.7	1.2	4.1	0.2	2000.4	1.8	1.9	
36	47.3	1.3	4.3	0.2	1997.4	1.8		
40	42.9	1.1	4.2	0.2	1994.0	1.9	1.8	
50	22.2	0.6	4.0	0.2	1990.5	1.9	1.9	
70	20.0	0.5	2.4	0.1	1986.3	2.1	1.9	
90	13.0	0.5	2.0	0.1	1981.5	2.3		
110	9.3	0.5	2.0	0.1	1975.6	2.8	1.8	
130	5.9	0.4	2.1	0.1	1966.9	3.6	1.8	
150	3.3	0.5	2.1	0.1	1954.5	4.9	1.8	
170	1.3	0.3	2.2	0.1	1939.8	7.3		
180	0.8	0.5	1.9	0.1	1922.9	11.8		
200	0.0	0.4	2.0	0.1	1902.2	19.8		
**December 2010 PCB06**								
2	67.6	2.3	11.6	1.2	2010.9	1.6	1.3	95.8
4	66.9	1.5	5.0	0.6	2010.9	1.6	1.2	90.1
6	67.1	1.3	5.7	0.6	2010.7	1.6	1.2	6.4
10	57.5	0.8	2.6	0.3	2009.3	1.7	1.2	**Pre-2010**
12	60.2	1.1	3.5	0.4	2005.9	1.7	1.2	6.3–8.6
14	55.6	1.1	3.4	0.5	2003.8	1.7	1.2	
16	56.0	1.1	2.7	0.5	2002.0	1.8	1.2	
18	49.1	1.0	3.9	0.5	2000.1	1.8	1.3	
20	46.1	0.9	3.5	0.4	1998.3	1.8	1.3	
30	34.4	0.8	4.1	0.4	1990.7	2.0	1.4	
38	30.2	0.8	3.1	0.4	1983.3	2.2	1.5	
42	28.4	0.8	3.7	0.4	1979.9	2.3	1.5	
52	20.3	0.8	3.5	0.4	1970.5	2.6	1.4	
62	15.8	0.5	2.8	0.3	1962.1	2.9	1.3	
72	12.3	0.6	3.1	0.3	1951.8	3.4	1.3	
82	10.0	0.7	3.9	0.4	1940.8	4.0	1.4	
92	6.9	0.5	3.3	0.4	1929.0	4.7	1.5	
105	2.2	0.3	1.8	0.2	1920.2	5.2	1.4	
115	2.3	0.3	1.8	0.2	1915.3	5.4	1.7	
135	2.0	0.2	2.1	0.2	1902.9	6.2	1.5	
155	1.3	0.2	2.0	0.2	1887.2	7.4		
**February 2011 PCB06**								
2	69.2	1.2	9.6	0.5	2011.1	1.0	1.9	12.1
6	55.3	1.0	4.6	0.3	2009.8	1.0	1.3	95.8
10	51.1	1.0	5.4	0.3	2008.3	1.0	1.2	90.1
14	54.9	0.9	4.1	0.2	2006.8	1.0	1.2	6.9
18	78.4	1.1	3.5	0.2	2004.7	1.0	1.2	**Pre-2010**
26	65.2	1.0	3.4	0.2	1999.2	1.0	1.2	6.3–8.6
34	63.8	1.0	4.4	0.3	1993.5	1.1	1.2	
50	47.8	0.9	4.0	0.2	1978.9	1.2	1.2	
66	30.2	0.7	4.0	0.2	1960.8	1.4	1.3	
75	15.9	0.3	2.5	0.1	1954.1	1.6	1.3	
95	6.6	0.2	2.3	0.1	1936.7	2.0	1.4	
125	3.3	0.2	2.8	0.1	1912.8	2.6	1.5	
155	2.0	0.2	3.0	0.1	1885.0	3.5	1.5	
